# Ethyl 2-(3-chloro-2-pyridyl)-5-oxopyrazolidine-3-carboxylate

**DOI:** 10.1107/S1600536809020789

**Published:** 2009-06-06

**Authors:** Hai-Jun Tan, Hai-Bing He, Ming Xia, Xiang-Ning Zhang, Hong-Jun Zhu

**Affiliations:** a Department of Applied Chemistry, College of Science, Nanjing University of Technology, Nanjing 210009, People’s Republic of China; b Jiangsu Pesticide Research Institute Co Ltd, Nanjing 210036, People’s Republic of China

## Abstract

In the mol­ecule of the title compound, C_11_H_12_ClN_3_O_3_, the five membered ring adopts an envelope conformation. In the crystal structure, inter­molecular N—H⋯O hydrogen bonds link the mol­ecules into centrosymmetric dimers.

## Related literature

For the synthetic procedure, see: Lahm *et al.* (2007 ▶). For bond-length data, see: Allen *et al.* (1987 ▶). 
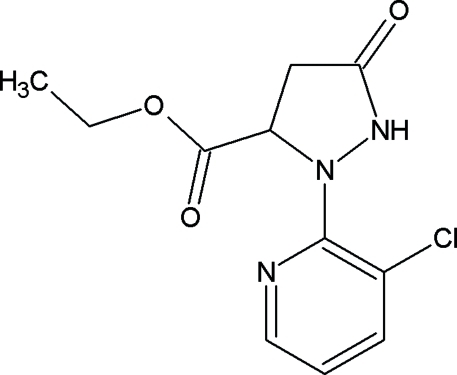


## Experimental

### 

#### Crystal data

C_11_H_12_ClN_3_O_3_
*M* *_r_* = 269.69
Orthorhombic, 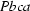

*a* = 15.488 (3) Å
*b* = 10.009 (2) Å
*c* = 16.249 (3) Å
*V* = 2518.9 (8) Å^3^
*Z* = 8
Mo *K*α radiation
μ = 0.31 mm^−1^
*T* = 298 K
0.30 × 0.20 × 0.10 mm

#### Data collection

Enraf–Nonius CAD-4 diffractometer
Absorption correction: ψ scan (North *et al.*, 1968 ▶) *T*_min_ = 0.913, *T*_max_ = 0.970
4453 measured reflections
2273 independent reflections
1635 reflections with *I* > 2σ(*I*)
*R*_int_ = 0.029
3 standard reflections frequency: 120 min intensity decay: none

#### Refinement

*R*[*F* ^2^ > 2σ(*F* ^2^)] = 0.043
*wR*(*F* ^2^) = 0.110
*S* = 1.03
2273 reflections
164 parameters
H-atom parameters constrained
Δρ_max_ = 0.29 e Å^−3^
Δρ_min_ = −0.23 e Å^−3^

### 

Data collection: *CAD-4 Software* (Enraf–Nonius, 1985 ▶); cell refinement: *CAD-4 Software*; data reduction: *XCAD4* (Harms & Wocadlo, 1995 ▶); program(s) used to solve structure: *SHELXS97* (Sheldrick, 2008 ▶); program(s) used to refine structure: *SHELXL97* (Sheldrick, 2008 ▶); molecular graphics: *SHELXTL* (Sheldrick, 2008 ▶); software used to prepare material for publication: *SHELXTL*.

## Supplementary Material

Crystal structure: contains datablocks I, global. DOI: 10.1107/S1600536809020789/hk2697sup1.cif

Structure factors: contains datablocks I. DOI: 10.1107/S1600536809020789/hk2697Isup2.hkl

Additional supplementary materials:  crystallographic information; 3D view; checkCIF report

## Figures and Tables

**Table 1:** Hydrogen-bond geometry (Å, °)

*D*—H⋯*A*	*D*—H	H⋯*A*	*D*⋯*A*	*D*—H⋯*A*
N1—H1*A*⋯O2^i^	0.86	2.14	2.910 (3)	149

Symmetry code: (i) 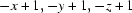.

**Fig. 1.**
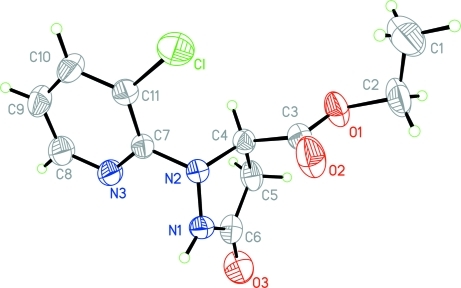
The molecular structure of the title molecule, with the atom-numbering scheme. Displacement ellipsoids at the 50% probability level.

**Fig. 2.**
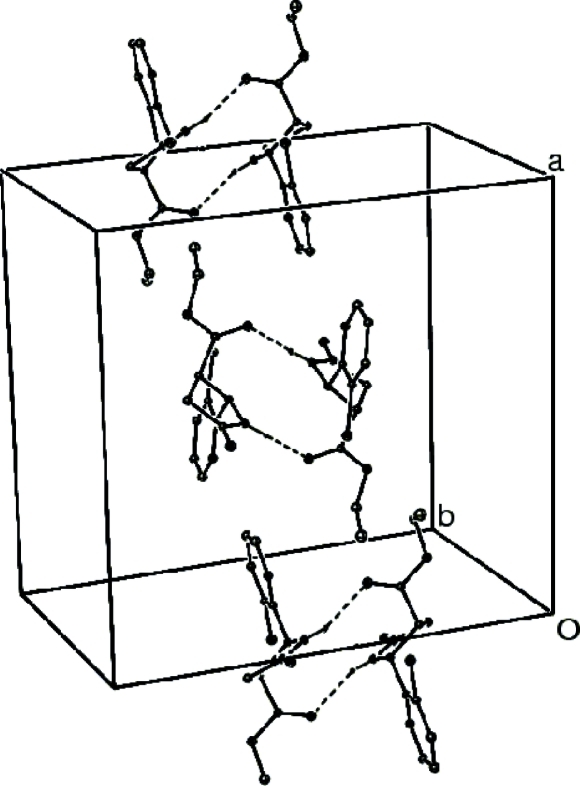
A partial packing diagram of the title compound. Hydrogen bonds are shown as dashed lines.

## supplementary crystallographic information

### Comment

The title compound is one of the most important intermediates used for the synthesis of Rynaxypyre, a new insecticidal anthranilic diamide as a potent and selective ryanodine receptor activator (Lahm *et al.*,  2007). We report herein the crystal structure of the title compound.

In the molecule of the title compound (Fig 1), the bond lengths (Allen *et al.*,  1987) and angles are within normal ranges. Ring B (N3/C7-C11) is, of course, planar. Ring A (N1/N2/C4-C6) adopts envelope conformation with atom C4 displaced by -0.375 (3) Å from the plane of the other ring atoms.

In the crystal structure, intermolecular N-H···O hydrogen bonds (Table 1) link the molecules into centrosymmetric dimers (Fig. 2), in which they may be effective in the stabilization of the structure.

### Experimental

The title compound was synthesized according to a literature method (Lahm *et al.*,  2007). Crystals suitable for X-ray analysis were obtained by slow evaporation of an ethanol solution.

### Refinement

H atoms were positioned geometrically, with N-H = 0.86 Å (for NH) and C-H = 0.93, 0.98, 0.97 and 0.96 Å for aromatic, methine, methylene and methyl H, respectively, and constrained to ride on their parent atoms, with U_iso_(H) = xU_eq_(C,N), where x = 1.5 for methyl H and x = 1.2 for all other H atoms.

### Crystal data

**Table:**

C_11_H_12_ClN_3_O_3_	*F*(000) = 1120
*M**_r_* = 269.69	*D*_x_ = 1.422 Mg m^−^^3^
Orthorhombic, *P**b**c**a*	Mo *K*α radiation, λ = 0.71073 Å
Hall symbol: -P 2ac 2ab	Cell parameters from 25 reflections
*a* = 15.488 (3) Å	θ = 0.9–1.0°
*b* = 10.009 (2) Å	µ = 0.31 mm^−^^1^
*c* = 16.249 (3) Å	*T* = 298 K
*V* = 2518.9 (8) Å^3^	Block, colorless
*Z* = 8	0.30 × 0.20 × 0.10 mm

### Data collection

**Table:**

Enraf–Nonius CAD-4 diffractometer	1635 reflections with *I* > 2σ(*I*)
Radiation source: fine-focus sealed tube	*R*_int_ = 0.029
graphite	θ_max_ = 25.3°, θ_min_ = 2.5°
ω/2θ scans	*h* = 0→18
Absorption correction: ψ scan (North *et al.*, 1968)	*k* = 0→12
*T*_min_ = 0.913, *T*_max_ = 0.970	*l* = −19→19
4453 measured reflections	3 standard reflections every 120 min
2273 independent reflections	intensity decay: none

### Refinement

**Table:**

Refinement on *F*^2^	Secondary atom site location: difference Fourier map
Least-squares matrix: full	Hydrogen site location: inferred from neighbouring sites
*R*[*F*^2^ > 2σ(*F*^2^)] = 0.043	H-atom parameters constrained
*wR*(*F*^2^) = 0.110	*w* = 1/[σ^2^(*F*_o_^2^) + (0.0473*P*)^2^ + 0.824*P*] where *P* = (*F*_o_^2^ + 2*F*_c_^2^)/3
*S* = 1.03	(Δ/σ)_max_ < 0.001
2273 reflections	Δρ_max_ = 0.29 e Å^−^^3^
164 parameters	Δρ_min_ = −0.23 e Å^−^^3^
0 restraints	Extinction correction: *SHELXL*, Fc^*^=kFc[1+0.001xFc^2^λ^3^/sin(2θ)]^-1/4^
Primary atom site location: structure-invariant direct methods	Extinction coefficient: 0.0065 (7)

### Special details

**Table:**

Geometry. All e.s.d.'s (except the e.s.d. in the dihedral angle between two l.s. planes) are estimated using the full covariance matrix. The cell e.s.d.'s are taken into account individually in the estimation of e.s.d.'s in distances, angles and torsion angles; correlations between e.s.d.'s in cell parameters are only used when they are defined by crystal symmetry. An approximate (isotropic) treatment of cell e.s.d.'s is used for estimating e.s.d.'s involving l.s. planes.
Refinement. Refinement of *F*^2^ against ALL reflections. The weighted *R*-factor *wR* and goodness of fit *S* are based on *F*^2^, conventional *R*-factors *R* are based on *F*, with *F* set to zero for negative *F*^2^. The threshold expression of *F*^2^ > σ(*F*^2^) is used only for calculating *R*-factors(gt) *etc*. and is not relevant to the choice of reflections for refinement. *R*-factors based on *F*^2^ are statistically about twice as large as those based on *F*, and *R*- factors based on ALL data will be even larger.

### Fractional atomic coordinates and isotropic or equivalent isotropic displacement parameters (Å^2^)

**Table:**

	*x*	*y*	*z*	*U*_iso_*/*U*_eq_	
Cl	0.45523 (4)	0.14184 (7)	0.43566 (5)	0.0629 (3)	
O1	0.30427 (10)	0.43898 (19)	0.31272 (11)	0.0561 (5)	
O2	0.34478 (11)	0.4578 (2)	0.44373 (11)	0.0597 (5)	
O3	0.55616 (12)	0.7407 (2)	0.33051 (13)	0.0667 (6)	
N1	0.54947 (12)	0.55100 (18)	0.40815 (12)	0.0372 (5)	
H1A	0.5776	0.5814	0.4497	0.045*	
N2	0.51613 (11)	0.41865 (17)	0.40548 (11)	0.0328 (4)	
N3	0.66149 (12)	0.3566 (2)	0.37919 (13)	0.0456 (5)	
C1	0.1756 (2)	0.3232 (4)	0.3592 (2)	0.0876 (11)	
H1B	0.1163	0.3358	0.3745	0.131*	
H1C	0.2069	0.2863	0.4049	0.131*	
H1D	0.1789	0.2631	0.3133	0.131*	
C2	0.21401 (15)	0.4539 (3)	0.3359 (2)	0.0666 (9)	
H2A	0.1820	0.4915	0.2902	0.080*	
H2B	0.2096	0.5152	0.3820	0.080*	
C3	0.36204 (14)	0.4419 (2)	0.37295 (15)	0.0389 (6)	
C4	0.45184 (14)	0.4216 (2)	0.33772 (13)	0.0363 (5)	
H4A	0.4542	0.3381	0.3063	0.044*	
C5	0.48079 (15)	0.5388 (3)	0.28374 (15)	0.0471 (6)	
H5A	0.5154	0.5078	0.2378	0.056*	
H5B	0.4315	0.5878	0.2627	0.056*	
C6	0.53378 (15)	0.6246 (3)	0.34130 (15)	0.0434 (6)	
C7	0.58184 (14)	0.3194 (2)	0.39697 (13)	0.0332 (5)	
C8	0.72260 (16)	0.2612 (3)	0.37550 (18)	0.0565 (8)	
H8A	0.7790	0.2872	0.3639	0.068*	
C9	0.70658 (18)	0.1279 (3)	0.38781 (17)	0.0548 (7)	
H9A	0.7509	0.0655	0.3842	0.066*	
C10	0.62367 (17)	0.0884 (3)	0.40552 (16)	0.0490 (6)	
H10A	0.6104	−0.0013	0.4134	0.059*	
C11	0.56048 (14)	0.1858 (2)	0.41127 (15)	0.0389 (6)	

### Atomic displacement parameters (Å^2^)

**Table:**

	*U*^11^	*U*^22^	*U*^33^	*U*^12^	*U*^13^	*U*^23^
Cl	0.0487 (4)	0.0514 (4)	0.0885 (6)	−0.0128 (3)	0.0079 (4)	0.0135 (4)
O1	0.0322 (9)	0.0815 (14)	0.0546 (11)	0.0039 (9)	−0.0095 (8)	−0.0041 (10)
O2	0.0367 (10)	0.0944 (16)	0.0479 (11)	0.0071 (10)	0.0002 (8)	−0.0127 (11)
O3	0.0629 (12)	0.0508 (12)	0.0864 (16)	−0.0110 (10)	−0.0068 (10)	0.0283 (11)
N1	0.0399 (10)	0.0307 (10)	0.0411 (11)	−0.0008 (8)	−0.0056 (9)	−0.0020 (9)
N2	0.0301 (9)	0.0308 (10)	0.0374 (10)	0.0009 (8)	−0.0036 (8)	−0.0011 (8)
N3	0.0314 (10)	0.0402 (12)	0.0650 (14)	0.0003 (9)	0.0035 (10)	−0.0027 (10)
C1	0.0537 (19)	0.097 (3)	0.112 (3)	−0.0200 (19)	0.0081 (19)	−0.017 (2)
C2	0.0295 (13)	0.087 (2)	0.084 (2)	0.0059 (14)	−0.0081 (14)	−0.0055 (18)
C3	0.0339 (12)	0.0399 (14)	0.0429 (14)	0.0027 (10)	−0.0047 (11)	−0.0039 (11)
C4	0.0342 (11)	0.0409 (13)	0.0339 (12)	0.0046 (10)	−0.0039 (9)	−0.0051 (10)
C5	0.0389 (13)	0.0647 (17)	0.0377 (13)	0.0061 (12)	−0.0017 (10)	0.0091 (12)
C6	0.0364 (13)	0.0465 (16)	0.0474 (15)	0.0045 (11)	0.0034 (11)	0.0107 (12)
C7	0.0318 (11)	0.0340 (12)	0.0337 (12)	0.0022 (10)	−0.0027 (10)	−0.0002 (10)
C8	0.0323 (12)	0.0542 (18)	0.083 (2)	0.0046 (12)	0.0019 (13)	−0.0045 (15)
C9	0.0498 (16)	0.0481 (16)	0.0666 (19)	0.0201 (13)	−0.0077 (14)	−0.0037 (13)
C10	0.0581 (16)	0.0342 (13)	0.0548 (15)	0.0072 (13)	−0.0068 (13)	0.0069 (12)
C11	0.0379 (13)	0.0351 (13)	0.0439 (14)	−0.0019 (10)	−0.0037 (10)	0.0032 (11)

### Geometric parameters (Å, °)

**Table:**

Cl—C11	1.735 (2)	C2—H2A	0.9700
O1—C2	1.456 (3)	C2—H2B	0.9700
O1—C3	1.326 (3)	C3—C4	1.518 (3)
O2—C3	1.191 (3)	C4—C5	1.531 (3)
O3—C6	1.226 (3)	C4—H4A	0.9800
N1—N2	1.422 (2)	C5—C6	1.512 (4)
N1—C6	1.335 (3)	C5—H5A	0.9700
N1—H1A	0.8600	C5—H5B	0.9700
N2—C4	1.485 (3)	C7—C11	1.396 (3)
N2—C7	1.429 (3)	C8—C9	1.372 (4)
N3—C7	1.321 (3)	C8—H8A	0.9300
N3—C8	1.346 (3)	C9—C10	1.374 (4)
C1—C2	1.485 (4)	C9—H9A	0.9300
C1—H1B	0.9600	C10—C11	1.385 (3)
C1—H1C	0.9600	C10—H10A	0.9300
C1—H1D	0.9600		
			
C3—O1—C2	117.0 (2)	C3—C4—H4A	110.1
N2—N1—H1A	122.5	C5—C4—H4A	110.1
C6—N1—N2	115.02 (19)	C6—C5—C4	103.85 (18)
C6—N1—H1A	122.5	C6—C5—H5A	111.0
N1—N2—C7	113.08 (17)	C4—C5—H5A	111.0
N1—N2—C4	104.33 (16)	C6—C5—H5B	111.0
C7—N2—C4	114.82 (17)	C4—C5—H5B	111.0
C7—N3—C8	117.8 (2)	H5A—C5—H5B	109.0
C2—C1—H1B	109.5	O3—C6—N1	126.0 (2)
C2—C1—H1C	109.5	O3—C6—C5	127.1 (2)
H1B—C1—H1C	109.5	N1—C6—C5	106.8 (2)
C2—C1—H1D	109.5	N3—C7—C11	121.9 (2)
H1B—C1—H1D	109.5	N3—C7—N2	119.3 (2)
H1C—C1—H1D	109.5	C11—C7—N2	118.7 (2)
O1—C2—C1	111.1 (2)	N3—C8—C9	123.8 (2)
O1—C2—H2A	109.4	N3—C8—H8A	118.1
C1—C2—H2A	109.4	C9—C8—H8A	118.1
O1—C2—H2B	109.4	C8—C9—C10	118.6 (2)
C1—C2—H2B	109.4	C8—C9—H9A	120.7
H2A—C2—H2B	108.0	C10—C9—H9A	120.7
O2—C3—O1	124.3 (2)	C9—C10—C11	118.2 (2)
O2—C3—C4	126.0 (2)	C9—C10—H10A	120.9
O1—C3—C4	109.70 (19)	C11—C10—H10A	120.9
N2—C4—C3	109.73 (17)	C10—C11—C7	119.7 (2)
N2—C4—C5	104.11 (18)	C10—C11—Cl	120.06 (19)
C3—C4—C5	112.46 (19)	C7—C11—Cl	120.25 (17)
N2—C4—H4A	110.1		
			
C6—N1—N2—C7	108.8 (2)	C4—C5—C6—O3	−164.6 (2)
C6—N1—N2—C4	−16.6 (2)	C4—C5—C6—N1	13.3 (2)
C3—O1—C2—C1	−85.7 (3)	C8—N3—C7—C11	−0.3 (3)
C2—O1—C3—O2	−1.2 (4)	C8—N3—C7—N2	176.9 (2)
C2—O1—C3—C4	178.6 (2)	N1—N2—C7—N3	−10.9 (3)
N1—N2—C4—C3	−97.1 (2)	C4—N2—C7—N3	108.6 (2)
C7—N2—C4—C3	138.54 (19)	N1—N2—C7—C11	166.3 (2)
N1—N2—C4—C5	23.4 (2)	C4—N2—C7—C11	−74.1 (3)
C7—N2—C4—C5	−100.9 (2)	C7—N3—C8—C9	1.1 (4)
O2—C3—C4—N2	2.4 (3)	N3—C8—C9—C10	−0.5 (4)
O1—C3—C4—N2	−177.44 (18)	C8—C9—C10—C11	−0.9 (4)
O2—C3—C4—C5	−113.0 (3)	C9—C10—C11—C7	1.7 (4)
O1—C3—C4—C5	67.2 (2)	C9—C10—C11—Cl	−178.6 (2)
N2—C4—C5—C6	−22.5 (2)	N3—C7—C11—C10	−1.1 (4)
C3—C4—C5—C6	96.2 (2)	N2—C7—C11—C10	−178.3 (2)
N2—N1—C6—O3	179.8 (2)	N3—C7—C11—Cl	179.22 (18)
N2—N1—C6—C5	1.8 (3)	N2—C7—C11—Cl	2.0 (3)

### Hydrogen-bond geometry (Å, °)

**Table:**

*D*—H···*A*	*D*—H	H···*A*	*D*···*A*	*D*—H···*A*
N1—H1A···O2^i^	0.86	2.14	2.910 (3)	149

Symmetry codes: (i) −*x*+1, −*y*+1, −*z*+1.
